# Fresh Water

**Published:** 1886-01

**Authors:** 


### HOME DEPARTMENT.

**Fresh Water.**—By this is meant the natural supplies of sweet water nearly free from mineral substances, and wholly free from organic matter. Natural fresh water is superior to all others for household use, owing to its impregnation with atmospheric air, and in some degree, with infinitesimal particles of lime, which render it slightly hard, but more wholesome, and with carbonic acid, which causes it to sparkle. These properties are all condusive to health.

Many persons believe that melted snow or ice waters are the healthiest, but it is the exclusion of air, lime and carbonic acid from distilled snow and ice water, which deprives them of the invigorating properties of fresh water, and the best of all fresh water is that procured from good artesian wells, which oftentimes extend to a very great depth.

